# Lowering Pulmonary Wedge Pressure after Heart Transplant: Pulmonary Compliance and Resistance Effect

**DOI:** 10.5935/abc.20150083

**Published:** 2015-09

**Authors:** Nádia Moreira, Rui Baptista, Susana Costa, Fátima Franco, Mariano Pêgo, Manuel Antunes

**Affiliations:** 1 Hospitais da Universidade de Coimbra. Rua Cidade de Santos, 66/67, 1º Dto, 3000-112 Coimbra, Monte Formoso. Postal Code 3000-112, Coimbra, Beira Litoral – Portugal; 2 Faculdade de Medicina da Universidade de Coimbra – Portugal

**Keywords:** Heart Transplantation, Pulmonary Wedge Pressure / physiology, Lung Compliance, Vascular Resistance

## Abstract

**Background:**

Right ventricular (RV) afterload is an important risk factor for post-heart transplantation (HTx) mortality, and it results from the interaction between pulmonary vascular resistance (PVR) and pulmonary compliance (CPA). Their product, the RC time, is believed to be constant. An exception is observed in pulmonary hypertension because of elevated left ventricular (LV) filling pressures.

**Objective:**

Using HTx as a model for chronic lowering of LV filling pressures, our aim was to assess the variations in RV afterload components after transplantation.

**Methods:**

We retrospectively studied 159 patients with right heart catheterization before and after HTx. The effect of Htx on hemodynamic variables was assessed.

**Results:**

Most of the patients were male (76%), and the mean age was 53 ± 12 years. HTx had a significant effect on the hemodynamics, with normalization of the LV and RV filling pressures and a significant increase in cardiac output and heart rate (HR). The PVR decreased by 56% and CPA increased by 86%. The RC time did not change significantly, instead of increasing secondary to pulmonary wedge pressure (PWP) normalization after HTx as expected. The expected increase in RC time with PWP lowering was offset by the increase in HR (because of autonomic denervation of the heart). This effect was independent from the decrease of PWP.

**Conclusions:**

The RC time remained unchanged after HTx, notwithstanding the fact that pulmonary capillary wedge pressure significantly decreased. An increased HR may have an important effect on RC time and RV afterload. Studying these interactions may be of value to the assessment of HTx candidates and explaining early RV failure after HTx.

## Introduction

Heart transplantation (HTx) is the treatment of choice for patients with end-stage heart failure (HF)^1^. The candidate's eligibility for HTx is strongly determined by the severity of pre-existing pulmonary hypertension (PH). PH may be secondary to the elevation of left ventricular (LV) filling pressures; however, it may also be associated with the presence of pulmonary vascular disease. While the former is completely reversible upon HTx, the latter may have a fixed component and lead to detrimental effects on right ventricular (RV) function. Indeed, an increased RV afterload is probably the most important risk factor for early post-HTx morbidity^2-5^.

RV hydraulic afterload is the result of the combined effects of a steady/fixed component, defined as the pulmonary vascular resistance (PVR), and a pulsatile/oscillatory component, usually reflected by pulmonary arterial compliance (CPA)^6^. The dynamic interaction between PVR and CPA determines the RV afterload, and their product (the RC time) is determined to be constant whether in health or disease, and before or after therapeutic interventions^7-10^.

A notable exception to this constant inverse relationship between CPA and PVR is observed in PH because of elevated LV filling pressures^11^. Elevated pulmonary capillary wedge pressure (PCWP) decreases the RC time because it simultaneously (i) increases pulmonary arterial stiffening (therefore, lowering CPA), and (ii) lowers the transpulmonary gradient (TPG) (therefore lowering PVR). Consequently, the proportional decrease in CPA is more than the corresponding increase in PVR^12^. Using HTx as a model for a chronic and definitive reduction in LV filling pressures, we aimed to assess the variations in several RV afterload components (PVR, CPA, and RC time) before and after surgery.

## Methods

### Study population

The study was approved by our institutional review board. We included two populations: a large, unselected population of patients that had undergone a right heart catheterization (RHC), defined as cohort 1, and a population of HTx patients, defined as cohort 2.

Cohort 1 included a retrospective cohort of 1797 consecutive patients who received an RHC for suspected PH or assessment of valvular disease severity between 2006 and 2011 in our hospital.

Cohort 2 included all patients who underwent HTx in our center and enrolled in a prospective, systematic follow-up program that included an RHC at least 6 months prior and 1 year after HTx. The pre-HTx RHC assesses eligibility and the absence of a high PVR that precludes HTx. The 1-year post-HTx RHC is performed during the scheduled endomyocardial biopsy (EMB) and coronary angiogram. From November 2003 to August 2013, a total of 236 HTx were performed. Of these, 30 patients died before the scheduled EMB; therefore, RHC data were not available for these patients. Of the remaining 201 patients, complete hemodynamic data with cardiac output were available for 159 patients.

### Right heart catheterization procedures

All RHC procedures were performed at rest, supine, via the femoral vein using fluoroscopic guidance, and were obtained by a clinical or interventional cardiologist specializing in HF. All pressure tracings were manually reviewed by the advanced HF physician and the surgeon. Patients were on optimal medical therapy for HF. Pressure measurements were obtained using fluid-filled 7F balloon-tipped catheters at end-expiration and registered in a working station.

The following hemodynamic parameters were measured or calculated: PCWP; mean, systolic, and diastolic pulmonary arterial pressures (mPAP, sPAP, and dPAP) and systemic arterial pressure; LV end diastolic pressure; heart rate (HR); pulmonary pulse pressure (PP); CO (calculated by the Fick method); stroke volume (SV), (obtained by dividing CO by HR); and right atrial pressure.

### Pulmonary Vascular Resistance, Compliance, and RC time

PVR was calculated using the equation [(mPAP - PCWP)/CO] and expressed in mmHg·s·mL^-1^. When PCWP tracings were of poor quality or were absent, LV end diastolic pressure was used instead. CPA was estimated by dividing the SV by the corresponding increase in the pulmonary artery pressure [(PP) = SV/PP], expressed in mL·mmHg^-1^. The RC time is calculated by PVR · CPA, expressed in seconds.

### Statistical analysis

Continuous data are presented as mean (standard deviation). All hemodynamic parameters were dichotomized according to data from the literature. Curve fits (linear or nonlinear) were generated, and statistical analysis was performed using regression. Comparisons between the cohorts were assessed using ANOVA; comparisons before and after HTx were performed using paired samples t-tests. Statistical significance was set at a level of p < 0.05. Commercially available statistical software packages were used (SPSS 13.0 and STATA 12.0). Graphs were drawn using GraphPad Prism 5.0 and STATA 12.0.

## Results

### Patient characteristics

Our study population consisted of two cohorts. Demographic and hemodynamic data are summarized in Table 1.

**Table 1:** Demographic and hemodynamic characteristics

	**Total population (n = 1797)**	**Pre-HTx (n = 159)**	**Post-HTx (n = 159)**	**p value**
Male gender, n (%)	1063 (59.2)	121 (76.1)	-	-
Age (years)	61 (14)	53 (12)	-	-
HR (min^-1^)	77 (16)	75 (17)	84 (12)	< 0.001
sPAP (mmHg)	44 (20)	46 (16)	31 (8)	< 0.001
dPAP (mmHg)	17 (9)	20 (8)	11 (5)	< 0.001
mPAP (mmHg)	28 (13)	30 (10)	20 (5)	< 0.001
PA pulse pressure (mmHg)	27 (14)	26 (10)	20 (6)	< 0.001
RA pressure (mmHg)	8 (5)	8 (6)	6 (3)	< 0.001
PCWP mean pressure (mmHg)	17 (9)	20 (8)	11 (5)	< 0.001
LV end diastolic pressure (mmHg)	18 (8)	20 (9)	15 (5)	< 0.001
TPG (mmHg)	11 (9)	10 (5)	9 (4)	0.041
CO (Lmin^-1^)	4.3 (1.6)	3.5(1.0)	5.8 (1.8)	< 0.001
SV (mL)	57 (23)	48 (17)	70 (23)	< 0.001
SBP, mean (mmHg)	89 (17)	75 (11)	98 (13)	< 0.001
Mixed venous saturation (%)	67 (9)	61 (8)	71 (8)	< 0.001
PVR (mmHgSmL^-1^)	0.19 (0.21)	0.18 (0.11)	0.10 (0.06)	< 0.001
CPA (mLmmHg^-1^)	2.7 (2.2)	2.1 (1.2)	3.9 (1.9)	< 0.001
RC time (seconds)	0.33 (0.18)	0.32 (0.17)	0.33 (0.14)	0.581

HTx: heart transplantation; HR: heart rate; Spap: systolic pulmonary arterial hypertension; dPAP: diastolic pulmonary arterial hypertension; mPAP: mean pulmonary arterial hypertension; PA: pulmonary artery; RA: right atrial; PCWP: pulmonary capillary wedge pressure; LV: left ventricle; TPG: Transpulmonary gradient; CO: cardiac output; SV: stroke volume; SBP: systolic blood pressure; PVR: pulmonary vascular resistance; CPA: pulmonary arterial compliance.

### Cohort 1

**Figure 1:**
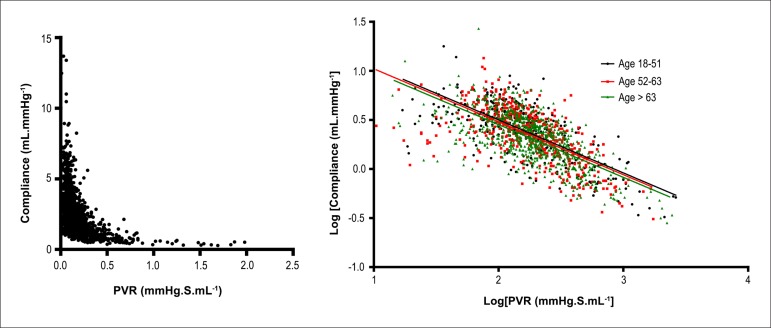
Left panel. An inverse hyperbolic relationship was found between pulmonary vascular resistance (PVR) and pulmonary arterial compliance (CPA) in cohort 1 patients. Right panel. Effect of age on the relationship between log[PVR] and log[CPA] in cohort 1, with the participants divided into three age groups. There is no significant difference between the slopes of the regression lines (p = 0.996).

Cohort 1 patients were mostly men (60%), and the mean age was 61 (14) years. Most patients had PH [mPAP 28 (13) mmHg]. The mean PVR was 0.19 (0.21) mmHg·s·mL^-1^, and the mean CPA was 2.7 (2.2) ml·mmHg^-1^. The mean RC time was 0.33 (0.18) seconds. We found an inverse relationship between PVR and CPA, as expected (Figure 1, left panel).

**Figure 2:**
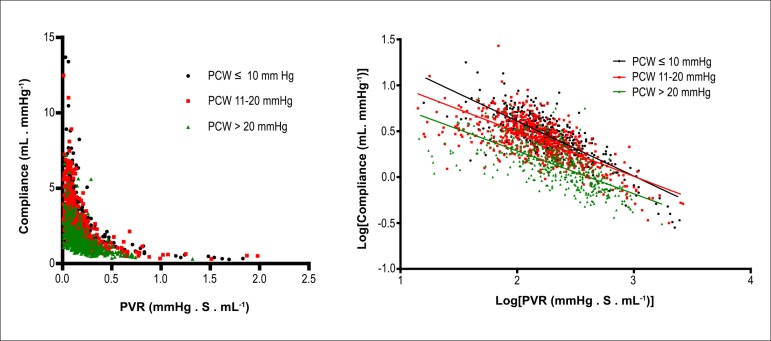
Upper left panel. Effect of pulmonary capillary wedge pressure (PCWP) on the relationship between pulmonary vascular resistance (PVR) and pulmonary arterial compliance (CPA) in cohort 1 patients, with PCWP divided into three subgroups. Upper right panel. Log[PVR] versus log[CPA]. There is a significant difference between the slopes of the regression lines (p < 0.001).

Age was expected to have an effect on the PVR-CPA relationship, as blood vessels tend to become less compliant over the years, similar to the systemic circulation. However, after grouping the patients according to age, we found that it had no impact on the PVR-CPA relationship (Figure 1, right panel). In contrast, PCWP had a significant effect on the PVR-CPA relationship, with higher PCWP leading to lower CPA for each level of PVR (Figure 2).

**Figure 3:**
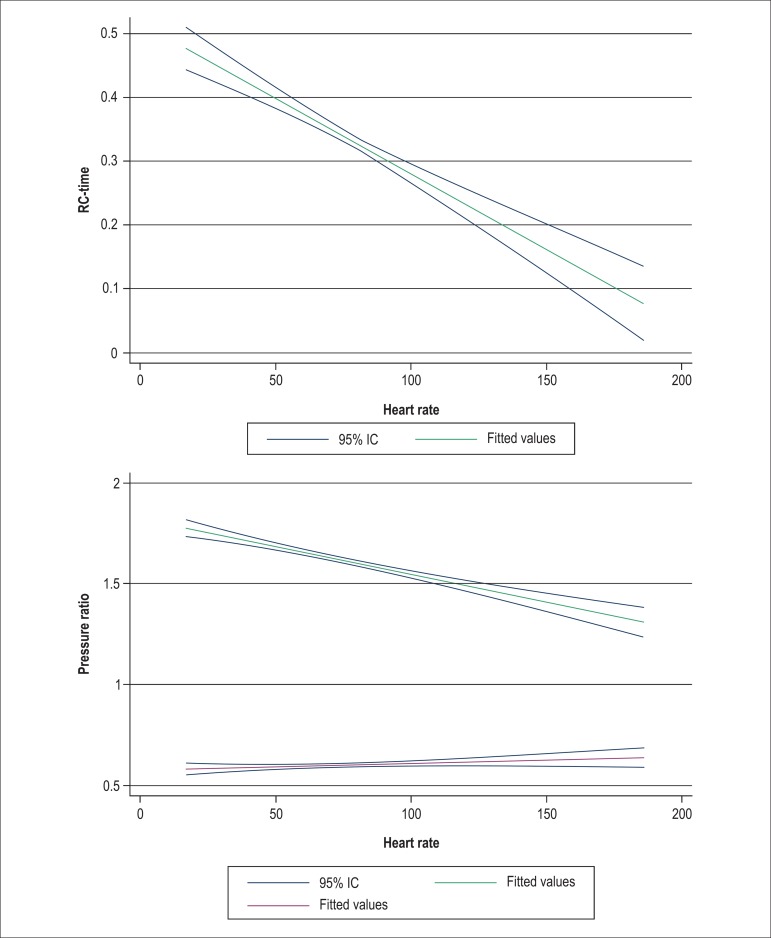
Left panel. Effect of heart rate (in bpm) on the RC time (in seconds) in cohort 1, with 95% confidence intervals. For the linear regression, there is a significant association between an increase in heart rate and a decrease in RC time (p < 0.001). Right panel. Effect of heart rate on the relationship between systolic and mean pulmonary arterial pressures (sPAP/mPAP: K_sys_) (upper line, blue), and diastolic and mean pulmonary arterial pressures (dPAP/mPAP: K_dia_) (lower line, green) in cohort 1 patients, with 95% confidence intervals. The heart rate clearly affects the pressure ratios, thus demonstrating that RC time is not constant when the heart rate is changing.

PCWP is not the only variable that affects the RC time; HR can also influence this variable^15^. We found a continuous effect of HR on RC time; a lower RC time is expected with a faster HR (Figure 3, left panel). This is further emphasized in Figure 3 (right panel): a lower HR is also related to a change in the relationships sPAP/mPAP (K_sys_) and dPAP/mPAP (K_dia_). A fixed RC time would yield a fixed relationship; as shown in Figure 3, there is a divergence of the ratios K_sys_ and K_dia_ as the HR lowers, which suggests that the RC time changes with HR. If the RC time was constant with varying HR, the lines would be parallel.

### Cohort 2

HTx patients had a mean age of 53 (12) years, and were mostly male. As expected, HTx had a very significant effect on the hemodynamics. CO increased by >2 L·min^-1^, and LV and RV filling pressures returned to normality. PVR decreased significantly to 0.10 mmHg·s·mL^-1^, whereas CPA rose by about 78% (to 3.9 mL·mmHg^-1^). Importantly, the HR increased by about 10 beats per minute, reflecting the autonomic denervation of the implanted heart (Table 1).

**Figure 4:**
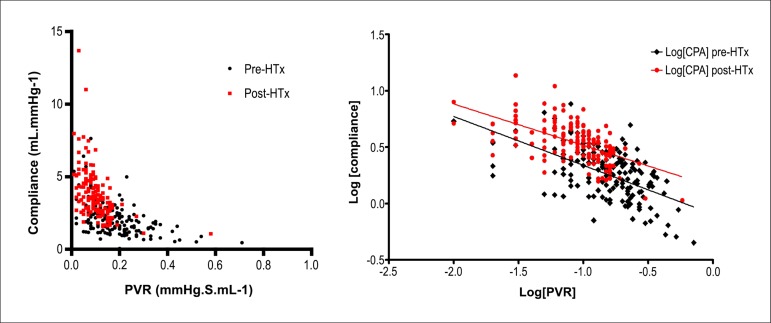
Effect of heart transplantation on the relationship between pulmonary vascular resistance (PVR) and pulmonary arterial compliance (CPA) before (black diamonds) and after heart transplantation (HTx) (red dots). Left panel. PVR versus CPA. Right panel. Log[PVR] versus log[CPA]. The slopes of the regression lines are not significantly different (p = 0.314).

We then examined the relationship between PVR and CPA before and after HTx. The RC time did not change significantly (0.32 to 0.33 seconds, p = 0.581), notwithstanding the fact that the PCWP dropped from 20 to 11 mmHg (p < 0.001) (Figure 4).

**Figure 5:**
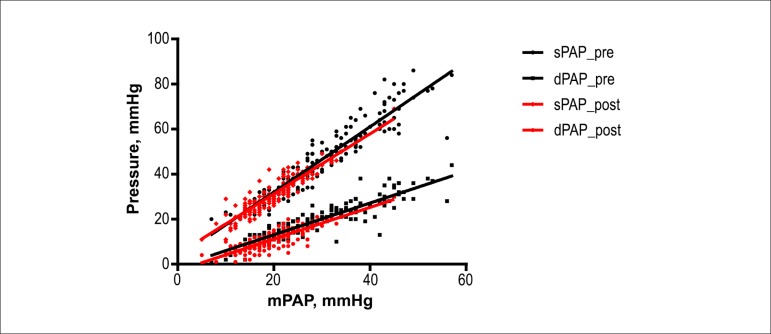
The relationship between the systolic and mean pulmonary arterial pressures (sPAP/mPAP: K_sys_) (p = 0.103), and diastolic and mean pulmonary arterial pressures (dPAP/mPAP: K_dia_) (p = 0.958) in HTx patients before (pre) and after intervention (post) is proportional, suggesting that RC time does not change with the procedure.

If the RC time remains constant after HTx, then the ratios between the pressures sPAP and mPAP (K_sys_), and dPAP and mPAP (K_dia_), should also be maintained. In fact, as we demonstrate in Figure 5, the slopes of K_sys_ and K_dia_ are similar before and after HTx, which suggests that the RC time has not changed with the surgical procedure.

**Figure 6:**
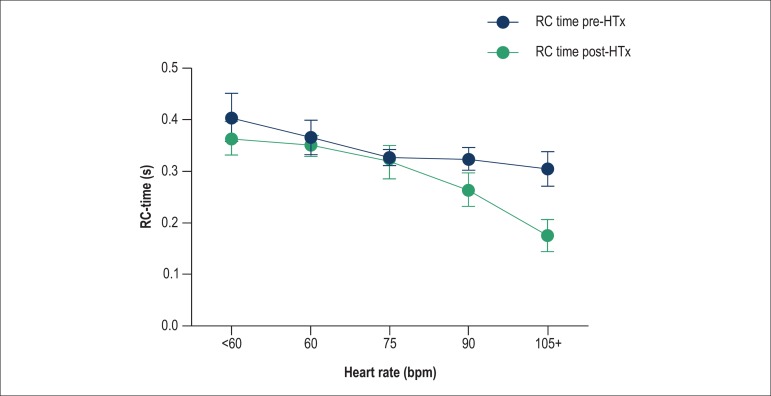
The RC time increases as the heart rate increases, offsetting the expected reduction of the former after heart transplantation. The difference between pre- and post-heart transplantation patients is more evident with increasing heart rate.

**Figure 7:**
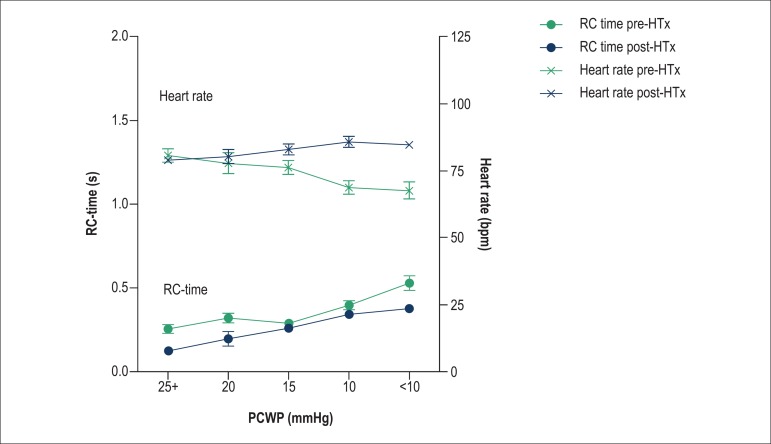
For the same levels of pulmonary capillary wedge pressure (PCWP), RC time (dots, lower panel) was found to be lower in the post-heart transplantation (HTx) patients (in red). The heart rate (crosses, upper panel) increased significantly after heart transplantation, but is not correlated with PCWP after heart transplantation.

If we control for HR on the impact of HTx on the RC time, when HR remains constant before and after HTx, the RC time would significantly increase (Figure 6). When simultaneously assessing the effect of PCWP on RC time and HR, it is clear that as the PCWP and HR lower, there is a corresponding increase in RC time, as expected. This is clearly seen in the pre-HTx data (black lines). However, this increase in RC time with decrease in PCWP is somewhat offset by the increase in HR that we observe after HTx (Figure 7).

## Discussion

This study demonstrates for the first time in an HTx population that there is an inverse relationship between CPA and PVR. It is well known that interventions aiming to lower LV pressures should increase the RC time. However, in our cohort of HTx patients, we did not observe any variation in RC time before and after HTx, which therefore does not reflect the anticipated effect of a decrease in LV filling pressures. This neutral variation may be caused either by a loss of arterial compliance along the extension into the pulmonary arteries due to long-standing elevated LV filling pressures or elevated HR secondary to heart denervation after transplantation. This results in a higher load on the RV than expected for such low PCWP.

Proximal pulmonary arterial obstruction has been known to augment wave reflection, which increases PP at any given level of mPAP. Increased wave reflection with disproportionate increase in sPAP relative to mPAP would add to the effects of increased pulmonary arterial stiffness, thus decreasing CPA and RC time, thereby increasing RV afterload at any level of PVR^12^.

### Why is the RC time constant?

Systolic PAP, dPAP, and mPAP are tightly correlated, with simple formulas to predict one from another that seem applicable to any type of PH (the basis of K_sys_ and K_dia_). However, it remains true only if CPA is predictable at any level of PVR; therefore, the RC time needs to remain constant^13^. Thus, if the RC time changes during or after an intervention, this association may not hold true. In our unselected population (cohort 1), there was a small, albeit significant, effect of HR on K_sys_ and K_dia_, which is clearly seen when the RC time is plotted against HR.

### Why does the RC time change with variations in PCWP?

The PVR-CPA relationship is sensitive to pulmonary venous pressure. This concept was first mentioned by Reuben et al^14^ in the context of increased left atrial pressure secondary to severe mitral stenosis. This author suggested that the disproportionate reduction of CPA relative to PVR was due to increased smooth muscle tone in the pulmonary arterial walls due to extremely high left atrial pressure^16^. This notion was recently revisited by Tedford et al^11^, who studied two populations with acute (exercise-induced) and chronic (drug- or HTx-mediated) variations in LV filling pressures. Tedford et al^11^ found that increasing PCWP progressively decreased the RC time, effectively enhancing RV pulsatile relative to the resistive load and suggesting that PCWP acts as the downstream pressure that amplifies peripheral pulse reflections^13^. This would augment sPAP (and PP), leading to a lower CPA for a given PVR, and thus, an increase in the TPG. As the TPG is calculated from mPAP, a disproportionate elevation of the sPAP would lead to an elevated TPG without pulmonary vasoconstriction or remodeling.

### Why did the RC time not increase as expected after HTx in our population?

The most intriguing result of our study was the fact that, although we observed a significant decrease in LV filling pressures in HTx recipients, PVR decreased more than the equivalent rise in CPA. Therefore, the RC time remained constant, when in fact it was expected to increase.

However, we also observed an elevation in the HR, which has an important role in the proportionality of pressures in the pulmonary circulation. As highlighted by Kind et al^13^, HR influences pressure ratios in that an increase in HR increases sPAP and decreases dPAP, thereby increasing PP. The HR not only reduces the heart period; it also increases PP, and therefore, contributes to a lower RC time. This lower-than-expected RC time after HTx may help explain why there is enlargement and some compromise of RV function after transplantation, even in patients without significant previous PH.

To our knowledge, the only study in the literature that has examined these relationships is the above mentioned study by Tedford et al^11^, in which 207 patients with a HF diagnosis were assessed at two distinct time points of RHC (one with a PCWP ≤ 10 mmHg and the other with a PCWP ≥ 20 mmHg). The authors state that some of these RHC were part of a HTx program, either pre- or post-operatively; however, there is no information regarding the precise number of pairs before/after transplantation. Importantly, the HR was the same (87 bpm) in both the “low PCWP” and “high PCWP” cohorts, which might be because of the small number of HTx patients. In this group, the authors found a significant decrease in RC time when the patient had a PCWP ≥ 20 mmHg^11^.

It was previously thought that the only exception to the constancy of the RC time was PH secondary to LV failure. In these patients, the RC time is decreased because increased pulmonary venous pressure results in a stiffer pulmonary arterial tree. The increased PCWP amplifies peripheral pulmonary arterial pulse reflection, thus augmenting sPAP. Hence, there is a decline in total CPA^11,13,15^. However, a recent study performed in an animal model of chronic thromboembolic PH demonstrated that exclusive proximal obstruction is another cause of decreased RC time; the study also showed an associated increase in PP and oscillatory arterial hydraulic work^12^. In contrast to previous report by de Perrot et al^16^ in 34 patients, Mackenzie Ross et al^17^ found a significant decrease in the RC time after pulmonary endarterectomy in 91 patients. The authors suggested that the lack of normalization of the RC time was due to the intervention on the tunica intima and media of the pulmonary artery, which affects the elastic properties of the blood vessel after surgery^17^. We suggest that the pulmonary vessels may retain some vascular tone (or loss of CPA) after long-standing exposure to elevated LV filling pressures, thereby altering the resistance-compliance relationship even after normalization of output pressures with HTx.

The critical role of clinically assessing the pulsatile components of RV afterload in HF patients has been highlighted in several reports. In a recent study of a cohort of HF patients, Pellegrini et al^18^ demonstrated that CPA is the single most important predictor of cardiovascular mortality, independent of PVR.

## Limitations

The retrospective nature of this study may have limited our access to clinical data. We used normal fluid-filled catheters for pressure assessments (and not high-fidelity pressure and flow measurements); however, this is the system that is currently used in most hemodynamic laboratories, and has been extensively tested in other reports.

## Conclusions

In our population of HTx patients, and in contrast to what was expected, the RC time did not change from pre-HTx values, although a very significant negative variation in LV filling pressures was observed after surgery. We believe that the higher HR observed after transplantation may be partially responsible for this finding. This may have affected the measurement of RV afterload in these patients.
